# Hepatic β-Oxidation and Regulation of Carnitine Palmitoyltransferase (CPT) I in Blunt Snout Bream *Megalobrama amblycephala* Fed a High Fat Diet

**DOI:** 10.1371/journal.pone.0093135

**Published:** 2014-03-27

**Authors:** Kang-Le Lu, Wei-Na Xu, Li-Na Wang, Ding-Dong Zhang, Chun-Nuan Zhang, Wen-Bin Liu

**Affiliations:** College of Animal Science and Technology, Nanjing Agricultural University, Nanjing, Jiangsu province, People’s Republic of China; Northeast Ohio Medical University, United States of America

## Abstract

High-fat diets may promote growth, partly through their protein-sparing effects. However, high-fat diets often lead to excessive fat deposition, which may have a negative impact on fish such as poor growth and suppressive immune. Therefore, this study investigated the effects of a fat-rich diet on the mechanisms of fat deposition in the liver. Three-hundred blunt snout bream (*Megalobrama amblycephala*) juveniles (initial mass 18.00±0.05 g) were fed with one of two diets (5% or 15% fat) for 8 weeks. β-Oxidation capacity and regulation of rate-limiting enzymes were assessed. Large fat droplets were present in hepatocytes of fish fed the high-fat diet. This observation is thought to be largely owing to the reduced capacity for mitochondrial and peroxisomal β-oxidation in the livers of fish fed the high-fat diet, as well as the decreased activities of carnitine palmitoyltransferase (CPT) I and acyl-CoA oxidase (ACO), which are enzymes involved in fatty-acid metabolism. Study of CPT I kinetics showed that CPT I had a low affinity for its substrates and a low catalytic efficiency in fish fed the high-fat diet. Expression of both CPT I and ACO was significantly down-regulated in fish fed the high-fat diet. Moreover, the fatty-acid composition of the mitochondrial membrane varied between the two groups. In conclusion, the attenuated β-oxidation capacity observed in fish fed a high-fat diet is proposed to be owing to decreased activity and/or catalytic efficiency of the rate-limiting enzymes CPT I and ACO, via both genetic and non-genetic mechanisms.

## Introduction

The use of fat-rich feeds has made fish farming more cost effective because protein is a relatively expensive source of energy [1]–[3]. Supplementing diets with fat enables protein to be “spared” for the synthesis of new tissue [4]–[6]. Indeed, increasing dietary lipid levels supports higher growth rates and spares dietary protein in some species. However, too much dietary lipid often leads to unwanted fat deposition in the liver, inducing a condition referred to as “fatty liver” [7]–[9]. Fatty liver is often considered in a negative light in cultured fish because it represents wasted energy; indeed, there is little point in supplying an energy-yielding nutrient that is simply deposited unused in tissues stores [10]. Furthermore, the health of fish may be affected by fatty liver, which often closely positively correlates with mortality [7], [11], [12]. Therefore, it is necessary to investigate the nutritional factors and mechanisms that affect the development of fatty liver.

It has generally been assumed that, in mammals, attenuated hepatic fatty acid (FA) β-oxidation is a common feature of fatty liver [13], [14]. According to Du et al. [6], impaired hepatic β-oxidation capacity also occurs when fish are fed fat-rich diets. To the best of our knowledge, few studies have looked at β-oxidation regulation. Carnitine palmitoyltransferase (CPT) I is considered to be the key regulatory enzyme in mitochondrial β-oxidation because it catalyzes the conversion of fatty acyl-CoAs into fatty acyl-carnitine molecules for entry into the mitochondrial matrix [15]. The regulation of CPT I is complex, including allosteric inhibition by malonyl-CoA [16], changes in the expression of the *CPT I* gene and transcription factors [17], and changes in the mitochondrial membrane composition [18], [19]. Estimating kinetic constants is critical to describe enzyme-catalyzed reactions [20]. However, it is not known how these regulatory mechanisms affect the activity and kinetics of CPT I in fish fed a high-fat diet. Evaluation of the major sites of lipid catabolism may provide further insight into the cause of excessive liver fat deposition in cultured fish.

Blunt snout bream (*Megalobrama amblycephala*) is an herbivorous freshwater fish native to China [21]. Due to its fast growth, tender flesh, and high disease resistance, this species has been widely favored for aquaculture in China [22]. However, comparable to a number of other commercially produced fishes, its artificial rearing is often associated with the occurrence of fatty liver, which correlates closely with a high rate of mortality or poor growth [9], [23]. Considering this, the present study evaluated hepatic FA β-oxidation and its regulation in blunt snout bream fed low- or high-fat diets. The result may have implications for our understanding of how fatty liver develops and may help to prevent metabolic diseases in cultured fish.

## Materials and Methods

### Ethic statement

Animal care and use were conducted in accordance with the Animal Research Institute Committee guidelines of Nanjing Agricultural University, China. This study was specifically approved by the Committee of Animal Research Institute, Nanjing Agricultural University, China.

### Experimental fish and feeding trial

Juvenile blunt snout bream was obtained from the fish hatchery of Wuhan (Hubei, China). The experiment was performed in a recirculating aquaculture system of laboratory. Prior to the experiment, fish were reared in several 250-l tanks (60 juveniles per tank) for 2 weeks to acclimate to the experiment conditions. After the acclimation period, 300 fish of similar size (average weight of 18.00±0.05 g; fork length: 9.50±0.07 cm) were randomly distributed into twelve 100-l tanks at the rate of 25 juveniles per tank. Water temperature, dissolved oxygen (DO), and pH were monitored daily. During the feeding period, fish were reared under the following conditions: water temperature, 25–27°C; DO, 5.0–6.0 mg/l; pH, 7.2–7.6; photoperiod, 12:12 h (dark: light). Fish were hand-fed to apparent satiation three times daily (08:00, 12:00, and 16:00 h) using two experimental diets (5 and 15% fat). Formulation and proximate composition of the experimental diets are presented in Table 1. Fatty acid composition of fish oil, soybean oil and the experimental diets are presented in Table 2. Feed offered was quantified daily for each aquarium of fish. Each treatment was tested in sextuplicate, and the trial lasted 8 weeks.

**Table 1:** Formulation and proximate composition of the experimental diets.

Ingredients (g/kg)	Diets		Proximate composition (g/kg)	Diets	
	Low-fat	High-fat		Low-fat	High-fat
Fish meal	150	150	Moisture	98.0	97.0
Casein	150	150	Crude protein	311	313
Soybean meal	200	200	Crude lipid	49.0	147
Corn starch	250	250	Crude fiber	138	41.0
α-starch	50.0	50.0	Ash	78.0	80.0
Fish oil	19.0	69.0	Carbohydrate a	326	323
Soybean oil	19.0	69.0	Energy b	14.9	18.8
Cellulose	104	4.00			
Calcium biphosphate	18.0	18.0			
Premix c	10.0	10.0			
Carboxymethyl cellulose	30.0	30.0			

a Carbohydrate (nitrogen-free extract) was calculate by difference (1000-moisture - crude protein - crude lipid – ash - crude fiber).

b Energy (ΚJ/g diet) = (%crude protein×23.6)+(%crude lipids×39.5)+ (%carbohydrates×17.3).

c Premix supplied the following minerals (g/kg) and vitamins (IU or mg/kg): CuSO_4_·5H_2_O, 2.0g; FeSO_4_·7H_2_O, 25g; ZnSO_4_·7H_2_O, 22g; MnSO_4_·4H_2_O, 7 g; Na_2_SeO_3_, 0.04 g; KI, 0.026 g; CoCl_2_·6H_2_O, 0.1 g; Vitamin A, 900000IU; Vitamin D, 200000IU; Vitamin E, 4500 mg; Vitamin K_3_ , 220 mg; Vitamin B_1_, 320 mg; Vitamin B_2_, 1090 mg; Niacin, 2800 mg; Vitamin B_5_, 2000 mg; Vitamin B_6_, 500 mg; Vitamin B_12_, 1.6 mg; Vitamin C, 5000 mg; Pantothenate, 1000 mg; Folic acid, 165 mg; Choline, 60000 mg.

**Table 2:** Fatty acid composition of fish oil, soybean oil and the diets.

Fatty acids (%)	Fish oil a	Soybean oil	Diets b
C14:0	6.26	0.07	3.08
C16:0	21.50	10.39	17.70
C18:0	5.12	4.89	4.96
C20:0	0.68	0.44	0.43
∑ SFA	33.56	15.79	26.17
C16:n-9	6.80	0.09	3.22
C18:n-9	14.58	25.14	21.52
C20:n-9	2.27	0.30	1.14
∑ MUFA	23.65	25.53	25.87
C18:2n-6	3.65	52.78	31.50
C18:3n-3	2.69	5.42	3.84
C20:5n-3 (EPA)	9.77	-	3.81
C22:5n-3	0.98	-	0.46
C22:6n-3 (DHA)	11.92	-	4.61
∑ PUFA	29.01	58.2	44.21

a Provided by Coland Feed Industry Co., Ltd (Wuhan, China).

b The fatty acids composition of the two diets are similar.

### Sample collection

At the end of the feeding trial, fish were starved overnight prior to sampling. Then, ten fish per tank were sampled and immediately euthanized by 100 mg/l MS-222 (tricaine methanesulfonate; Sigma, USA). Liver was removed (placed on ice) and then stored at –70°C until analysis. Additionally, the liver samples for the histology observations were fixed in the relevant buffer.

### Histology study

Samples for transmission electron microscopy observation were fixed in 2.5% glutaraldehyde for 24 h, post-fixed in 1% osmium tetroxide (OsO_4_) for 1 h, and stored at 4°C. Sections were embedded in epoxy resin Epon812, cut into70-nm-thick sections with a RMC PowerTome XL microtome, stained with uranyl acetate and lead citrate, and examined under a Hitachi H-7650 (Hitachi, Tokyo, Japan) transmission electron microscope.

### Mitochondrial and peroxisomal β-oxidation

Mitochondrial and peroxisomal β-oxidation was determined in postnuclear fractions as acid-soluble products using radiolabelled [1-^14^C] palmitate as a substrate, as described previously [6]. Livers (about 1 g) were homogenized in nine volume ice-cold sucrose medium (0.25 M sucrose in 10 nM HEPES buffer at pH 7.4, with and 1 mM EDTA) and postnuclear- fractions were prepared. Palmitate oxidation rates were measured at 28°C using two media as described by Frùyland et al. [24], the first allowing the total (mitochondrial and peroxisomal) activities to occur (13.2 mM HEPES (pH 7.3), 16.5 mM MgCl_2_, 82.5 mM KCl, 13.2 mM dithiothreitol, 6.6 mM ADP, 0.2 mM NAD^+^, 100 μM-CoA and 0.7 mM EDTA), the second allowing the peroxisomal activity only (the medium only differing by the presence of 73 mM antimycin and 10 mM rotenone to block the respiratory chain). The palmitate oxidation was measured with 115 μM [1-^14^C] palmitate supplemented with 1.2 mM L-carnitine. The samples were incubated for 30 min at room temperature then reactions were stopped by addition of 1.5 M KOH; Fatty acid-free bovine serum albumin (BSA, 100 mg/ml) was added to the suspension in order to bind unoxidized substrates and then 4 M HClO_4_ was added to precipitate unoxidized substrates bound to BSA. The total solution was then centrifuged at 1880 *g* for 15 min. Aliquots of 200 μl were transferred to a scintillation tube containing 4 ml of liquid scintillation cocktail and assayed for radioactivity in a LS6500 liquid scintillation analyser (Beckman, USA). Mitochondrial β-oxidation was obtained by subtracting the peroxisomal β-oxidation from the total β-oxidation.

### Acyl-CoA Oxidase Assay

The assay of Acyl-CoA oxidase (ACO) activity was based on the determination of H_2_O_2_, production, which was coupled to the oxidation of 2′,7′-dichlorofluorescine, essentially as described by Kjær et al. [25]. The oxidation of 2′,7′-dichlorofluorescine by hydrogen peroxide to 2′,7′-dichlorofluorescein was followed spectrophotometrically at 502 nm in a spectrophotometer. The reaction mixture contained 0.1 M Tris-HCl (pH 8.5), 0.05 M 2′,7′-dichlorofluorescine, 50 μM horseradish peroxidase, 0.015 mM FAD, 60 mg/ml BSA and 0.02% Triton-X 100, and was started with 60 μM palmitoyl-CoA. The reaction mixture contained about 100 μg protein in a total volume of 1 ml at 28°C.

### Isolation of liver mitochondria

The mitochondria were extracted according to Suarez and Hochachka [26]. Mitochondrial isolation buffer (MIB) consisted of 250 mM sucrose, 1 mM EDTA, 20 mM HEPES and 0.5 % bovine serum albumin (BSA) (pH 7.4). Liver samples (about 2 g) were carefully weighed and homogenized in nine volume ice-cold buffer. Then, homogenates were centrifuged at 800 *g* for 10 min at 4°C. The supernatant was centrifuged at 9, 000 *g* for 10 min at 4°C to obtain the mitochondrial pellet. The latter were resuspended in a small volume of the appropriate MIB lacking BSA. The resuspended homogenate was collected into a centrifuge tube and centrifuged again at 9, 000 *g* for 10 min at 4°C. The mitochondrial pellet was resuspended in an appropriate volume of MIB lacking BSA.

### Determination of CPT I activity and kinetics

CPT I activity was analyzed using the method of Bieber and Fiol [27], based on measurement of the initial CoA-SH formation by the 5, 5′-dithio-bis-(2-nitrobenzoic acid) (DTNB) reaction from palmitoyl-CoA by mitochondria samples with L-carnitine at 412 nm. Briefly, 50 μl buffer solution (containing 116 mM Tris, 2.5 mM EDTA, 2 mM DTNB, 0.2% Triton X-100, pH 8.0) and 50 μl mitochondria suspension were added to microcuvette. After 5 min preincubation at 28°C, 50 μl of 1 mM palmitoyl-CoA was added to the cuvettes. The reaction was then started by adding 5 μl of 1.2 mM L-carnitine solution, immediately followed by photometric measurement at 412 nm at 28°C. The CPT I activity was expressed as nmol CoA-SH produced/(min mg protein) at 28°C. Mitochondrial protein concentration was determined using Lowry et al.’s method [28].

For the kinetic studies, the ranges of substrate concentrations for carnitine were from 0.50 to 10 mM, and for palmitoyl-CoA from 0.02 to 0.60 mM. The enzymatic reaction was initiated by adding palmitoyl-CoA (100 μM) and carnitine (400 mM) to generate palmitoylcarnitine and incubated at 28°C. Analysis of the kinetic data was performed as described by Hofstee [29]. The values of the Michaelis-Menten constants (*K*_m_) and maximal reaction velocity (*V*_max_) were analyzed using a non-linear regression method described by the Michaelis-Menten equation. Lineweaver-Burk graphs [29] were drawn by using 1/v versus 1/[S] values. Catalytic efficiency, defined as an enzyme’s efficiency in transforming its substrate, was calculated by the ratio between maximum enzyme activity and *K*_m_ (*V*_max_/*K*_m_). The concentration of malonyl-CoA (M-CoA) to reduce the activity of M-CoA-sensitive CPT I activity by 50% (IC_50_) as determined, according to Morash et al. [30]. For IC_50_ assay, the ranges of concentrations for M-CoA were from 0.05 to 50 μM. All measurements were performed in duplicate.

### Analysis of malonyl-CoA (M-CoA) level in liver

M-CoA content in liver were determined using a method from Richards et al. [31]. M-CoA was extracted from 50 mg lyophilized liver in 15 times its weight of 0.5 M HClO_4_ containing 50 μM dithioerythritol and 10 mg/ml propionyl-CoA as an internal standard. After homogenization at 0°C, homogenates were centrifuged at 20, 000 g for 10 min at 4°C, and 200 μl of supernatant were transferred to a borosilicate vial adjusted to pH 3 using 4 M NaOH while being vortexed. Supernatants were transferred to autosample vials containing 20 μl of 1 M MOPS (pH 6.8), and final pH of the sample was determined using pH paper: pH was always <5. Autosampler vials were placed in a Waters 717 Plus autosampler (Waters, Missisauga, ON) at room temperature and M-CoA was separated using reverse-phase HPLC based on a method from Demoz et al. [32]. Briefly, 200 μl of the sample was injected onto a Zorbax ODS Rx C-18 column (25 cm×0.46 mm) (Agilent). An elution gradient, set up by a Waters Model 510 pump controller, was used to separate the CoA esters. Solvent A was 100 mM sodium phosphate and 75 mM sodium acetate in ultrapure deionized water (pH 4.2), and solvent B was the same as A except in 30% CH_3_CN. The gradient was as follows: 0 min, 90% A; 10 min, 60% A; 17.6 min, 10% A. Baseline condition was established again after 5 min of washing with 90% A. The elution was carried out at ambient temperature, and the flow rate was 1.5 ml/min. Absorbance measurements were made at 254 nm on a Lambda Max 481 LC spectrophotometer (Waters). Resulting peaks were manually identified by comparison of retention times to standards of known composition, and peaks were quantified by comparison with the internal standard.

### Assay of mitochondrial enzyme activities and membrane FA composition

For enzymatic analysis, the mitochondrial pellets were suspended in the isolation medium. Succinate dehydrogenase (SDH) was estimated with sodium succinate as substrate, according to the method of Philip et al. [33]. Na^+^-K^+^-ATPase activity was measured according to McCormick et al. [34]. Total superoxide dismutase (SOD) activity was measured with the commercial kit (Nanjing JianCheng Bioengineering Institute, China), according to Nakano [35]. Mitochondrial protein concentration was determined using Lowry et al.’s method [28]. Enzymatic activities were expressed in units (U) per mg mitochondrial protein. Thiobarbituric acid reactive substances (TBARS) were performed as described by Rueda-Jasso et al. [36], using a malondialdehyde (MDA) kit (Nanjing JianCheng Bioengineering Institute, China).

For FA composition analysis, mitochondrial total lipid were extracted by the method of Folch [37]. Then, FAs from samples were methylated using 0.5 mol/l NaOH in methanol for 30 min at 60°C and then esterified in 25% BF_3_ in methanol. FA methyl esters were then analyzed and quantified using a Shimadzu GC-201 gas chromatograph in a cross-linked 5% phenylmethyl silicone gum phase column (length 30 m; internal diameter 0.32 mm; film thickness 0.25 mm; N_2_ as the carrier gas), equipped with flame ionisation detection. The injector and detector temperatures were 250°C. The oven temperature was kept at 100°C for 3min, raised to 180°C at the rate of 10°C /min and then raised to 240°C at 3°C /min. FAs in the samples were identified by comparison of retention times with those of an authentic standard compounds mixture. Results are expressed as the percentage of each FA with respect to total FAs.

### Total RNA extraction, reverse transcription and real-time PCR

Total RNA was extracted from the liver tissue using RNAiso Plus (Takara Co. Ltd, Japan). RNA samples were treated by RQ1 RNase-Free DNase prior to RT-PCR (Takara Co. Ltd, Japan) to avoid genomic DNA amplification. cDNA was generated from 500 ng DNase-treated RNA using ExScript™ RT-PCR kit (Takara Co. Ltd, Japan), and the mixture consisted of 500 ng RNA, 2 μl buffer (5×), 0.5 μl dNTP mixture (10 mM each), 0.25 μl RNase inhibitor (40 U/μl), 0.5 μl dT-AP primer (50 mM), 0.25 μl ExScript™ RTase (200 U/μl), and DEPC H_2_O, with total volume up to 10 μl. The reaction conditions were as follows: 42°C for 40 min, 90°C for 2 min, and 4°C thereafter.

Real-time PCR was employed to determine mRNA levels based on the SYBR Green I fluorescence kit. Primer characteristics used for real-time PCR are listed in Table S1. Real-time PCR was performed in a Mini Option real-time detector (BIO-RAD, USA). The fluorescent quantitative PCR reaction solution consisted of 12.5 μl SYBR® premix Ex Taq™ (2×), 0.5 μl PCR forward primer (10 μM), 0.5 μl PCR reverse primer (10 μM), 2.0 μl RT reaction (cDNA solution), and 9.5 μl dH_2_O. The reaction conditions were as follows: 95°C for 3 min followed by 45 cycles consisting of 95°C for 10 s and 60°C for 20 s. The fluorescent flux was then recorded, and the reaction continued at 72°C for 3 min. The dissolution rate was measured between 65 and 90°C. Each increase of 0.2°C was maintained for 1 s, and the fluorescent flux was recorded. All amplicons were initially separated by agarose gel electrophoresis to ensure that they were of correct size. A dissociation curve was determined during the PCR program to make sure that specific products were obtained in each run. The gene expression levels were normalised towards the mean of the reference gene (β-actin). Normalised gene expressions of the low-fat group were set to 1, and the expression of each target gene for the high-fat group was expressed relative to low-fat group.

### Statistical analysis

Data were analyzed by SPSS ver. 16.0 for Windows software (SPSS, Chicago, IL). Student’s *t*-test was used to analyze differences between two treatments. The level of significance was set at *P*<0.05. All data were presented as means ± S.E.M. (standard error of the mean).

## Results

### Ultrastructure of the hepatocyte

**Figure 1:**
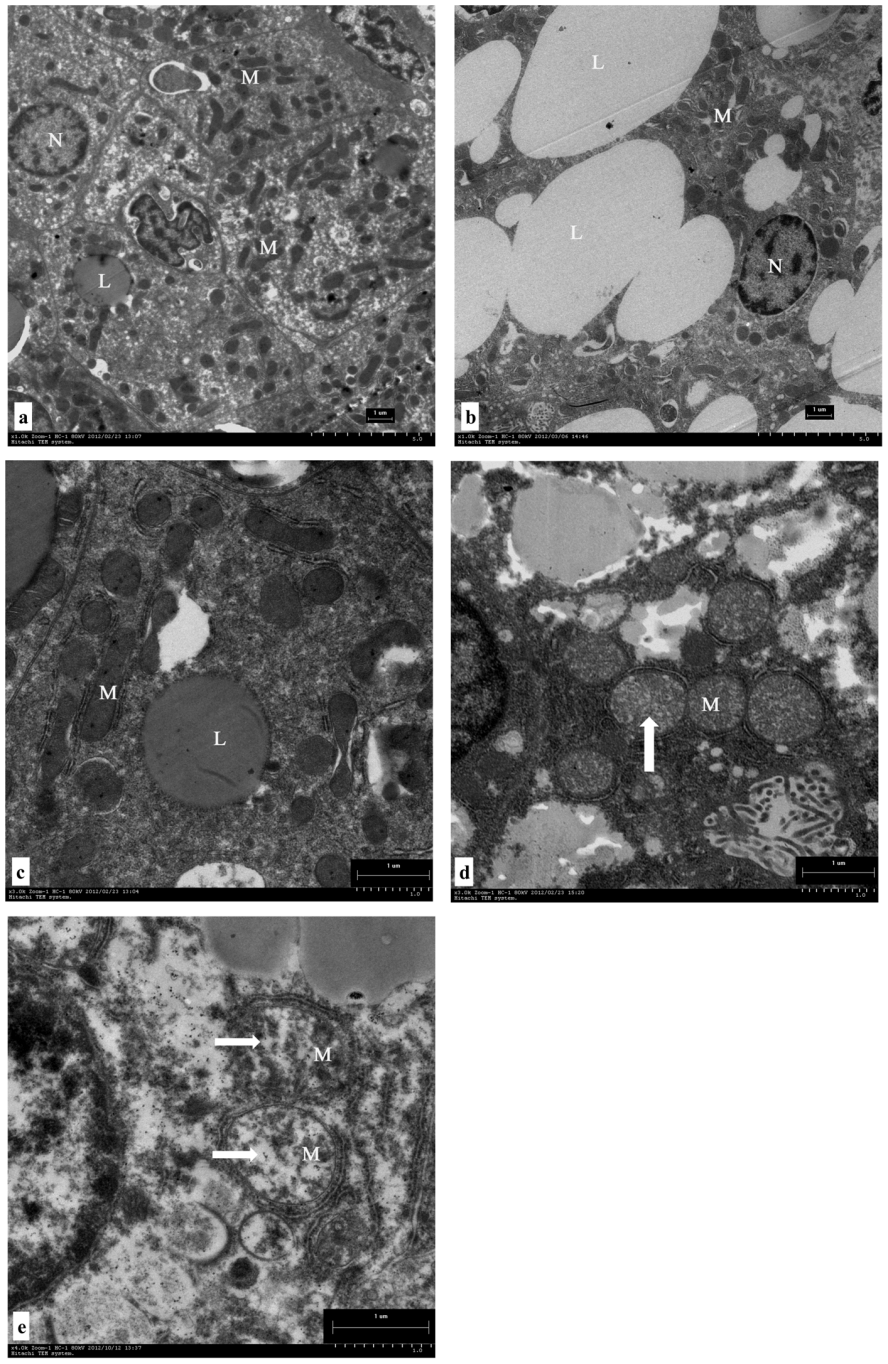
Transmission electron microscope images of blunt snout bream hepatocyte and mitochondrion ultrastructure: N (Nucleus), L (lipid droplet), M (mitochondrion). Photomicrographs and main findings: (a) hepatocytes of fish fed low-fat diet with normal structure; (b) hepatocytes presenting extensive intracellular lipid droplets of fish fed high-fat diet; (c) hepatocytes of fish fed low-fat diet displaying dark and slender mitochondria; (d, e) mitochondria showing highly hydropic changes (↑, →) of fish fed high-fat diet. Bar = 1 μm.

Livers of fish fed the low-fat diet had a normal ultrastructure. Hepatocytes had a large and ovoid nucleus that was centrally located with moderate cytoplasm (Fig. 1a) and a prominent nucleolus. Hepatocytes displayed dark and slender mitochondria (Fig. 1c). However, the livers of fish fed the high-fat diet exhibited several abnormalities. Hepatocytes exhibited many large and electron-dense fat droplets, some of which were even larger than the nucleus (Fig. 1b). These intracellular lipid droplets resulted in the displacement of the nucleus to the cell margin and loss of cytoplasm (Fig. 1b). The mitochondria altered metrical density with highly hydropic changes (Fig. 1d, e).

### Hepatic FA β-oxidation

**Figure 2:**
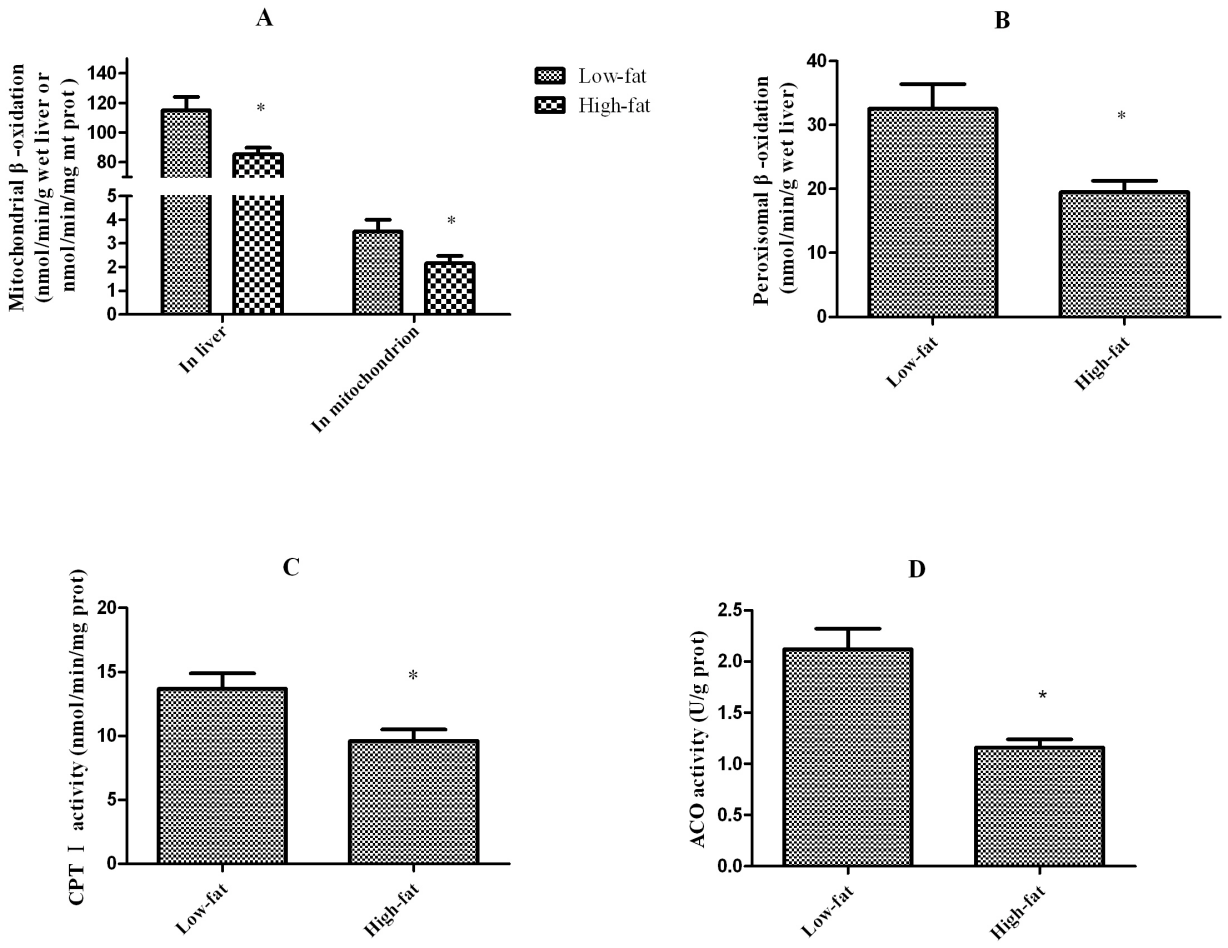
Parameters related to mitochondrial and peroxisomal β-oxidation in liver of blunt snout bream fed the experimental diets. (A) Mitochondrial β-oxidation in liver homogenate or mitochondrial-fraction. (B) Peroxisomal β-oxidation in liver. (C) CPT I activity in mitochondrial-fraction. (D) Acyl-CoA oxidase (ACO) activity in liver homogenate. Mean values and standard error (±S.E.M.) are present for each parameter (n = 6). ^*^, Significantly different from the fish fed control diet: *P*<0.05.

Parameters related to mitochondrial and peroxisomal β-oxidation in the liver are shown in Figure 2. The mitochondrial oxidation rate, which was measured from liver homogenate, was significantly lower (*P*<0.05) in fish fed the high-fat diet than in fish fed the low-fat diet (115±9.1 *vs.* 85.2±4.6 nmol/min/g wet liver). When expressed per milligram of the mitochondrial protein fraction, the oxidation rate was still significantly lower (*P*<0.05) in fish fed the high-fat diet than in fish fed the low-fat diet. CPT I activity, expressed per milligram of the mitochondrial protein fraction, was significantly lower (*P*<0.05) in fish fed the high-fat diet than in fish fed the low-fat diet (13.7±1.2 *vs.* 9.6 ±0.9 nmol/min/mg prot).

The peroxisomal FA oxidation rate, expressed per gram of liver homogenate, was also significantly lower (*P*<0.05) in fish fed the high-fat diet than in fish fed the low-fat diet (32.5±3.9 *vs.* 19.5 ±1.8 nmol/min/g wet liver). In addition, the activity of acyl-CoA oxidase (ACO), which is an enzyme involved in FA metabolism, was significantly lower (*P*<0.05) in fish fed the high-fat diet than in fish fed the low-fat diet (2.12±0.2 *vs.* 1.16 ±0.1 U/g prot).

### Kinetic parameters of CPT I

**Figure 3:**
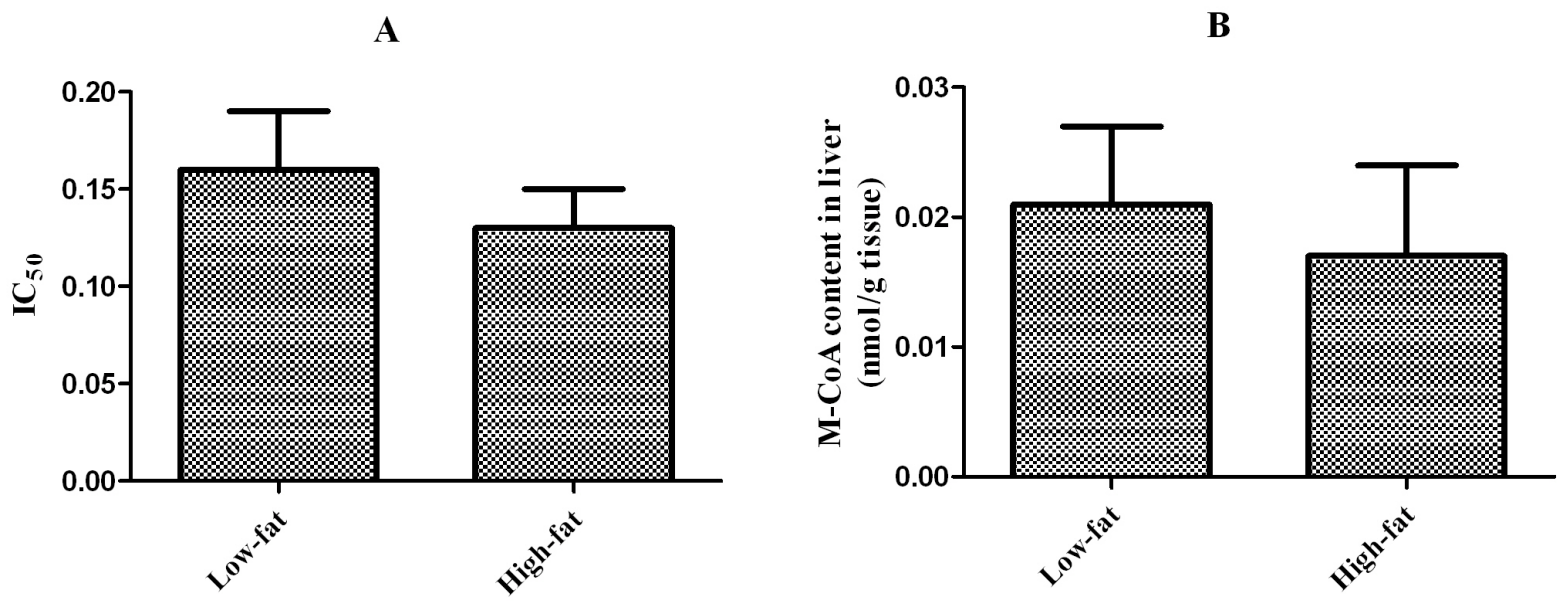
IC_50_:The concentration of malonyl-CoA (μM) to reduce the activity of CPT I by 50% (A); Malonyl-CoA (M-CoA) content in liver (B); Mean values and standard error (±S.E.M.) are present for each parameter (n = 6).

Several kinetic parameters of CPT I, namely, maximum rate (*V*_max_), Michaelis constant (*K_m_*), and catalytic efficiency, are presented in Table 3. When using carnitine and palmitoyl-CoA as substrates, CPT I *V*_max_ was significantly lower (*P*<0.05) in fish fed the high-fat diet than in fish fed the low-fat diet (12.7±0.5 *vs.* 15.3±0.9 nmolmin/mg/mt prot for carnitine, and 12.1±0.6 *vs.* 14.7±0.7 nmolmin/mg/mt prot for palmitoyl-CoA). *K*_m_ was significantly higher (*P*<0.05) in fish fed the high-fat diet than in fish fed the low-fat diet (2.76±0.1 *vs.* 1.69±0.07 mM for carnitine, and 93.1±7.3 *vs.* 68.9±6.2 μM for palmitoyl-CoA). Moreover, the catalytic efficiency of CPT I was significantly lower (*P*<0.05) in fish fed the high-fat diet than in fish fed the low-fat diet (4.60±0.5 *vs.* 9.05±0.6 for carnitine, and 0.14±0.02 *vs.* 0.22±0.03 for palmitoyl-CoA). The CPT I IC_50_ values and liver malonyl-CoA content did not significantly differ (*P* > 0.05) between fish fed the high-fat diet and those fed the low-fat diet (Figure 3).

**Table 3:** Kinetic analysis of CPT I in liver of blunt snout bream fed the experimental diets.

Parameters		Low-fat	High-fat
V_max_ (nmolmin/mg/mt prot)	For Carnitine	15.3±0.9	12.7±0.5^*^
	For Palmitoyl-CoA	14.7±0.7	12.1±0.6^*^
*K_m_*	For Carnitine (mM)	1.69±0.07	2.76±0.10^*^
	For Palmitoyl-CoA (μM)	68.9±6.2	93.1±7.3^*^
Catalytic efficiency	For Carnitine	9.05±0.6	4.60±0.5^*^
	For Palmitoyl-CoA	0.22±0.03	0.14±0.02^*^

Mean values and standard error (±S.E.M.) are present for each parameter (n = 6).

^*,^ Significantly different from the fish fed control diet: *P*<0.05.

### Mitochondria status

**Figure 4:**
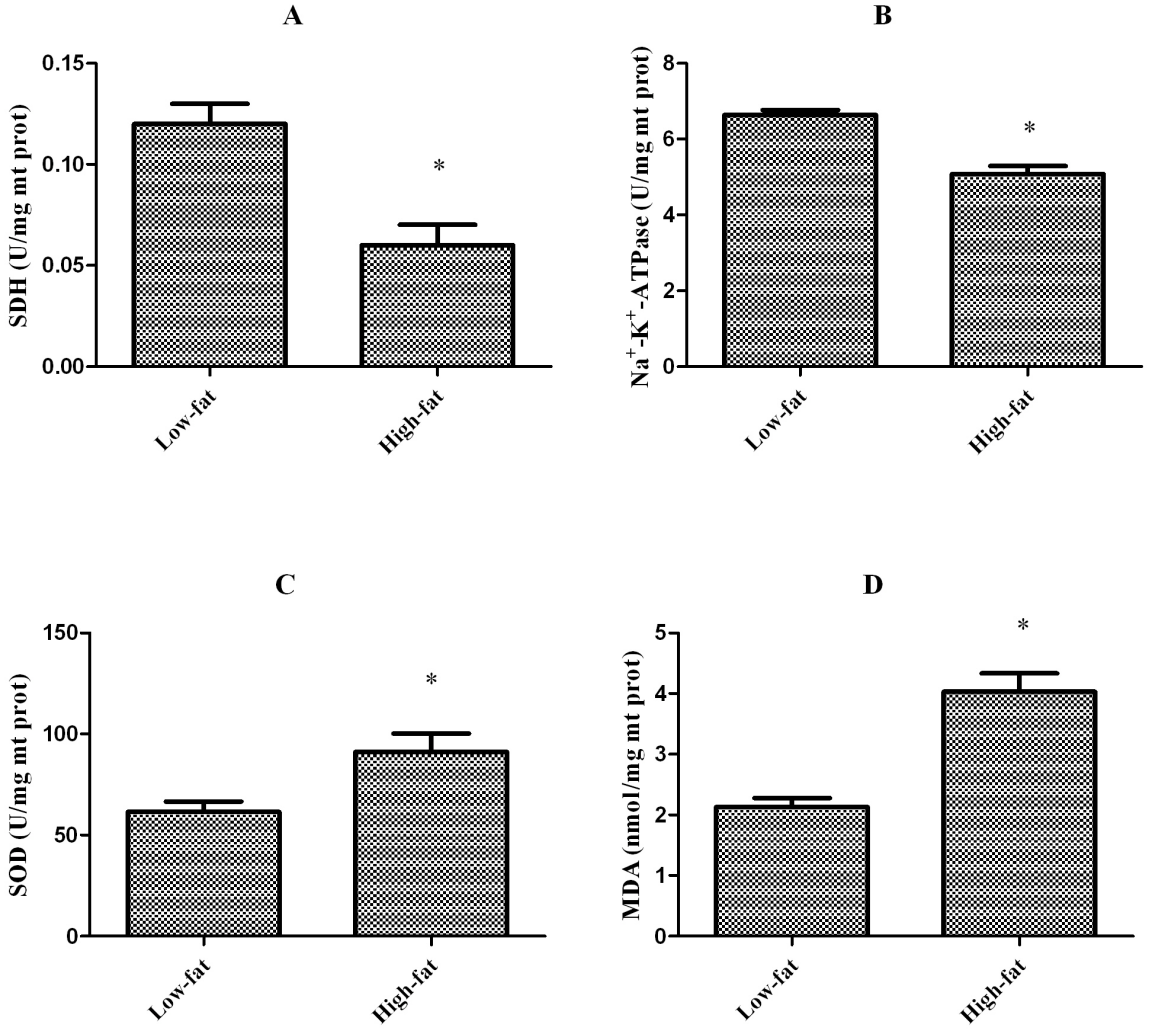
Mitochondria status parameters in blunt snout bream fed the experimental diets. (A) SDH activity in mitochondrial fraction. (B) Na^+^-K^+^-ATPase in mitochondrial fraction. (C) SOD activity in mitochondrial fraction. (D) MDA level in mitochondrial fraction. Mean values and standard error (±S.E.M.) are present for each parameter (n = 6). ^*^, Significantly different from the fish fed control diet: *P*<0.05.

Mitochondrial succinate dehydrogenase (SDH), Na^+^-K^+^-ATPase, superoxide dismutase (SOD) activities, and malondialdehyde (MDA) levels in liver are presented in Figure 4. SDH and Na^+^-K^+^-ATPase activities were significantly lower (*P*<0.05) in fish fed the high-fat diet than in fish fed the low-fat diet (0.06±0.01 *vs.* 0.12±0.02 U/mg mt prot for SDH, and 5.08±0.21 *vs.* 6.64±0.13 U/mg mt prot for Na^+^-K^+^-ATPase). However, mitochondrial SOD activity and the level of MDA were significantly higher (*P*<0.05) in fish fed the high-fat diet than in fish fed the low-fat diet (91.2±9.0 *vs.* 61.5±5.0 U/mg mt prot for SOD, and 4.03±0.30 *vs.* 2.13±0.15 nmol/mg mt prot for MDA).

The FA composition of the liver mitochondrial membrane is shown in Table 4. The levels of saturated fatty acid (SFA) and monounsaturated fatty acid (MUFA) did not significantly differ between fish fed a high-fat diet and those fed a low-fat diet (*P* > 0.05). However, levels of polyunsaturated fatty acids (PUFAs) (18: 3*n*-3, 20: 5*n*-3, and 22: 6*n*-3) were significantly higher (*P*<0.05) in fish fed the high-fat diet than in fish fed the low-fat diet. In addition, the percentages of the total *n*-3 PUFAs, very long-chain FAs, and the *n*-3/*n*-6 ratio were significantly higher (*P*<0.05) in fish fed the high-fat diet than in fish fed the low-fat diet.

**Table 4:** Fatty acid composition of mitochondrial membrane in liver of blunt snout bream fed the experimental diets.

Fatty acids (%)	Mitochondrial membrane	
	Low-fat	High-fat
C14:0	0.72±0.05	0.75±0.02
C16:0	17.1±0.39	16.7±0.16
C18:0	13.5±0.26	13.2±0.27
C20:0	0.18±0.01	0.20±0.02
∑ SFA	31.5±0.62	30.8±0.44
C16:n-9	1.82±0.03	1.39±0.03^*^
C18:n-9	15.1±0.20	14.1±0.21
C20:n-9	0.54±0.02	0.55±0.01
∑ MUFA	17.5±0.23	16.1±0.16
C18:2n-6	11.0±0.29	12.1±0.14
C18:3n-3	0.67±0.04	0.87±0.02^*^
C20:5n-3 (EPA)	2.74±0.13	3.78±0.10^*^
C22:5n-3	1.21±0.05	1.44±0.10
C22:6n-3 (DHA)	21.8±0.62	26.2±0.05^*^
∑ PUFA	37.4±0.83	44.4±0.23^*^
∑ n-3	26.4±0.70	32.3±0.24^*^
∑ n-6	11.0±0.29	12.1±0.14
∑ VLCFA a	23.0±0.66	27.6±0.13^*^
n-3/n-6	2.39±0.07	2.66±0.04^*^

Mean values and standard error (±S.E.M.) are present for each parameter (n = 6).

^*,^ Significantly different from the fish fed control diet: *P*<0.05.

a VLCFA: very long chain fatty acid.

### Gene expression

**Figure 5:**
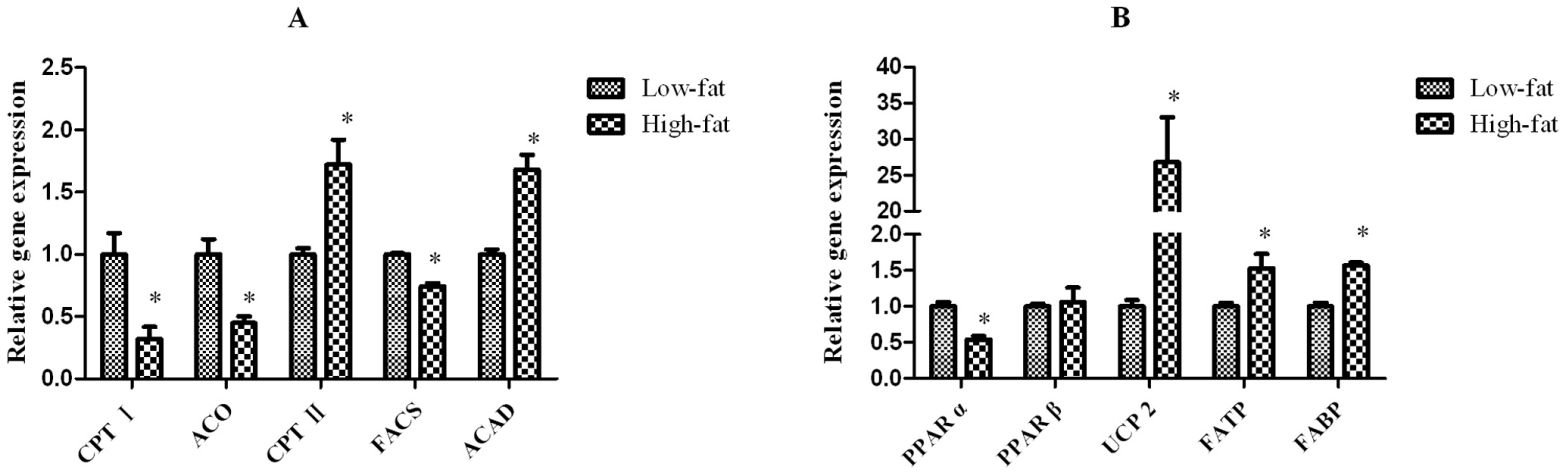
Relative gene expressions of lipid-related genes. (A) Genes involved in mitochondrial and peroxisomal β-oxidation (CPT I, ACO, CPT II, FACS and ACAD). (B) Genes involved in gene regulation (PPARs), fatty acid uptake and transport (FATP and FABP) and uncoupling protein (UCP 2). Mean values and standard error (±S.E.M.) are present for each parameter (n = 6). The values of the expression of the target genes are presented as relative to value of low-fat group (set to 1). Data were normalized by β-actin. ^*^, Significantly different from the fish fed control diet: *P*<0.05. *PPAR: peroxisome proliferatoractivated receptor; ACO: acyl-CoA oxidase; ACAD: acyl-CoA dehydrogenase; CPT I, II: carnitine palmitoyltransferase I, II; FACS: fatty acyl-CoA synthetase; FATP: fatty acid transport protein; FABP: fatty acid binding protein; UCP 2: uncoupling protein 2.*

The expression of genes involved in lipid metabolism is shown in Figure 5. The mRNA levels of fatty acyl-CoA synthetase (FACS), CPT I, and ACO was significantly lower (*P*<0.05) in fish fed the high-fat diet than in fish fed the low-fat diet. The expression of two peroxisome proliferator-activated receptor (PPAR) isoforms (α and β) differed. PPARα expression was significantly lower (*P*<0.05) in fish fed the high-fat diet than in fish fed the low-fat diet. By contrast, the expression of PPARβ did not significantly differ between the two groups. The mRNA levels of CPT II, acyl-CoA dehydrogenase, fatty acid binding protein (FABP), fatty acid transport protein (FATP), and uncoupling protein 2 (UCP 2) were significantly higher (*P*<0.05) in fish fed the high-fat diet than in fish fed the low-fat diet.

## Discussion

In recent years, there has been a trend to increase dietary lipid levels in commercial fish feed formulations to enhance protein sparing and to increase the growth of fish and farm productivity [3], [4], [6], [38]. However, the use of high-energy diets directly influences fat deposition in the liver, which, in fish, has implications for both health and product quality [23], [39]. The liver fulfills numerous functions, some of which are related to metabolism, detoxification, digestion, and excretion [3], [7]. Long-term lipid accumulation in hepatocytes induces liver dysfunction, which develops into microscopic changes and, eventually, macroscopic lesions. In the present study, ultrastructural examination of the liver showed that excessive accumulation of fat in the cytoplasm was generally accompanied by nuclear atrophy to a level such that the fish can be described as having a pathological liver. The location of the nucleus in a hepatocyte is often mentioned when describing the accumulation of lipid droplets [40]–[42]. Pathological accumulation of lipid is suggested when the nucleus does not occupy the center of the cell [3]. Moreover, fatty liver also closely correlates with the poor growth. Indeed, the weight gain of fish fed high-fat diet was significantly lower than that of fish fed the low-fat diet in this study (129% *vs* 175%; data not shown).

FA oxidation is important in liver lipid metabolism, especially when animals ingest high-fat diets [6]. When dietary lipid intake exceeds the capacity of the hepatic cells to oxidize FAs, large amounts of triglyceride are synthesized and deposited in vacuoles, leading to steatosis. In fish, β-oxidation, the main pathway of FA oxidation, occurs in mitochondria and peroxisomes [43]. The present data indicate that mitochondria are responsible for 80% of hepatic β-oxidation in blunt snout bream. By contrast, peroxisomes are responsible for 100% of hepatic β-oxidation in haddock (*Melanogrammus aeglefinus*) [44]. The physiological significance of this dominance of peroxisomal or mitochondrial β-oxidation may be an adaptation to the FA composition of the diet. In rat hepatocytes, treatment with a partially hydrogenated marine oil caused a 3-fold increase in β-oxidation of erucic acid (a peroxisomal substrate) compared with peanut oil treatment. Mammals and fish appear to regulate the mitochondrial β-oxidation enzymes in a similar manner. Malonyl-CoA (a FA synthesis product) inhibits mitochondrial β-oxidation by reducing the activity of CPT I. Fish fed a lipid-rich diet may exhibit increased β-oxidation activity owing to a reduced level of malonyl-CoA and an increased availability of FA substrates. However, in the present study, both mitochondrial and peroxisomal β-oxidation in the liver were significantly decreased in fish fed a high-fat diet. Therefore, hepatic lipid accumulation mainly occurred because the excess lipids that were consumed could not be oxidized. In addition, our previous study showed that a high-fat diet not only affected the lipid level, but also FA composition in the liver [9]. This previous study showed that SFA and MUFA levels are strongly reduced, while PUFA accumulates, in blunt snout bream fed a 15% fat diet [9]. SFA and MUFA in fish are preferentially used as oxidation substrates through the mitochondrial pathway, whereas this is not the case for PUFA [45]. Thus, it is predicted that decreased FA oxidation will not only affect lipid levels, but also the FA content of the liver.

In mitochondrial fat oxidation, CPT I is thought to be a major regulatory mechanism with numerous regulating factors, both genetic and non-genetic [30]. In the present study, the mRNA level of CPT I was significantly lower in fish fed a high-fat diet than in fish fed a low-fat diet. The reduced expression of CPT I partly accounts for its low activity. It is generally accepted that many of the enzymes that are involved in hepatic FA oxidation and metabolism are influenced by PPARs [46]. Expression of CPT I mRNA is thought to be influenced by the PPAR transcription factors because it contains a PPAR response element [17]. The mammalian PPAR isoforms (α, β, and γ) have also been identified in numerous fish species, but their functional roles are different [47], [48]. PPARα activates lipid catabolism by regulating the expression of target genes encoding enzymes involved in peroxisomal and mitochondrial β-oxidation of FAs, mainly in the liver [49], while PPARγ plays an important role in lipid accumulation and adipocyte differentiation [50]. In the present study, the high-fat diet attenuated PPARα gene expression, which may correlate with the down-regulation of CPT I. PPARα mRNA is generally up-regulated by a high-fat diet in mammals [51], [52], which is in contrast to the current study. In fish, the function of PPARs in lipid metabolism may be even more complicated because whole genome duplication events lead to multiple isoforms of PPARs [48], [53]. Furthermore, their expression may vary across tissues, making genomic and functional studies much more difficult in fish than in mammals [19]. CPT I may also be inhibited by malonyl-CoA, which is produced during the first step of de novo FA synthesis by acetyl-CoA carboxylase [54]. However, in the present study, liver malonyl-CoA levels did not differ significantly between the two groups.

In addition to CPT I, the number of mitochondria is thought to play a role in determining the fat oxidative capacity of a tissue [6], [30]. In grass carp (*Ctenopharyngodon idella*), the rate of mitochondrial FA oxidation per gram of liver tissue decreases following an increase in dietary lipid intake. This is not due to reduced CPT I activity but to a dramatic decrease in mitochondrial protein content per gram of liver tissue [6]. However, the influence of mitochondrial quantity on fat oxidation has received little attention in fish. The ultrastructure and membrane FA composition of mitochondria have been postulated to be strongly related to the metabolic activity of mitochondria [55]. According to the mitochondria structural data presented in this study, there were distinct differences between blunt snout bream fed a high-fat diet and those fed a low-fat diet. In fish fed a 15% fat diet, mitochondria had fewer cristae, less matrix, and altered metrical density with highly hydropic changes. These changes suggest that mitochondria were damaged by exposure to oxidative stress because reactive oxygen species (ROS) induce damage that impairs organelle integrity [56]. The observation that SOD activity and MDA levels are increased in fish fed a high-fat diet supports the suggestion that the mitochondria are damaged by oxidative stress. There is a considerable amount of information on how manipulating the dietary FA composition changes the FA content of the mitochondrial membrane in fish [19]. However, little is known about how mitochondrial membranes in fish respond to changes in dietary lipid intake. The present study shows that dietary fat content markedly influences mitochondrial membranes. The *n*-3 PUFA levels were significantly higher in fish fed the high-fat diet than in fish fed the low-fat diet, and an increase in *n*-3 PUFA levels in the diet is thought to increase CPT I activity via increasing mitochondrial membrane fluidity [57]. However, the present study did not detect this enhancing effect of *n*-3 PUFA on CPT I activity. It is important to note that PUFAs are prone to oxidative damage, which may negatively affect the function of CPT I because of its strong interaction with the outer mitochondrial membrane [58]. UCP 2 is an inner mitochondrial membrane protein that mediates proton leak by uncoupling fuel oxidation from adenosine triphosphate (ATP) synthesis [59]. Increased UCP 2 expression is thought to promote substrate disposal and limit mitochondrial ROS production by decreasing the redox pressure on the electron transport chain [59]. In the current study, hepatic UCP 2 expression dramatically increased in fish fed the high-fat diet, which implies high ROS production. Mitochondria play a central role in the energy metabolism of cells and provide most of the ATP by oxidative phosphorylation; thus, mitochondrial lesions impair energy metabolism in the cell. In the present study, SDH and Na^+^-K^+^-ATPase activities were lower in fish fed a high-fat diet than in fish fed a low-fat diet. These two enzymes play important roles in energy metabolism, and any abnormalities in these enzymes may indicate a metabolic disorder.

Estimating kinetic constants is critical to describe enzyme-catalyzed reactions [60]. In the present study, fish fed a high-fat diet had increased *K*_m_ and decreased *V*_max_ values for CPT I in the liver. Patterns of enzyme *V*_max_ values across tissues are useful to reveal differences in FA oxidation capacity [30]. Enzymatic-catalytic efficiency relates the total enzyme concentration to the interaction between the enzyme and its substrate [20]. *K*_m_ is defined as the substrate concentration at which the catalyzed reaction occurs at half its maximum velocity. A small *K*_m_ indicates that the enzyme requires only a small amount of substrate to become saturated. Hence, the maximum velocity is reached at relatively low substrate concentrations. By contrast, a large *K*_m_ indicates that high substrate concentrations are needed to achieve the maximum reaction velocity. In this study, the *K*_m_ of CPT I was significantly higher in fish fed the high-fat diet than in fish fed the low-fat diet. Thus, CPT I has a lower ‘affinity’ for FAs in fish fed a high-fat diet, which leads to a lower velocity of oxidation. In a previous study, the low hepatic lipid content in juvenile *Synechogobius hasta* fed with *trans*-10, *cis*-12 conjugated linoleic acid was thought to be owing to the increased affinity of CPT I for its substrates (low *K*_m_ value) and its increased catalytic efficiency [20]. The mechanisms underlying the differences in the *K*_m_ of CPT I between fish fed a low-fat diet and those fed a high-fat diet are unknown, but might be explained by the following two hypotheses. First, the difference in *K*_m_ between the two groups may be associated with the expression profiles of CPT I isoforms owing to the co-expression of multiple CPT I isoforms in the liver. In mammals, CPT Iβ has a higher *K*_m_ than CPT Iα for l-carnitine [61]. Zheng et al. reported that four CPT I isoforms are expressed at the mRNA level in the liver of *Pelteobagrus fulvidraco* [62]. Therefore, it is reasonable to speculate that the differences in the kinetic characteristics of CPT I might be related to the number of CPT I isoforms expressed. However, further experiments in blunt snout bream are needed to verify this. Second, CPT I strongly interacts with the outer mitochondrial membrane [58], and its kinetics are highly dependent on the physical properties of this membrane [63]. Altering the mitochondrial membrane FA composition alters the specificity, affinity, and malonyl-CoA sensitivity of CPT I in rats [55]. In the current study, the liver mitochondrial membrane composition was significantly changed by a high-fat diet. These changes have implications for the fluidity of the membrane, and could thereby potentially alter the kinetics of CPT I. One possible mechanism underlying changes in the kinetics of CPT I is that alterations in the interactions of CPT I with other (lipid and/or protein) membrane components causes a conformational change in the protein that particularly affects its acyl-CoA-binding site [63]. Moreover, our ultrastructural and biochemical findings suggest that fish fed a high-fat diet had mitochondrial lesions, which may also alter CPT I kinetics.

In the present study, peroxisomes significantly contributed (about 30%) to total β-oxidation. Short-chain FA are mainly oxidized within mitochondria, and long-chain FA, such as 22:6*n*-3, are very poor substrates for mitochondrial FA β-oxidation [25]. ACO is thought to catalyze the first rate-limiting step in peroxisomal β-oxidation [64]. In this study, decreased ACO activity in fish fed a high-fat diet was attributed to decreased peroxisomal β-oxidation. In addition to CPT I and ACO, many other enzymes may function in β-oxidation in fish [65]. FACS has been suggested to be an important regulator of β-oxidation in FA activation [66]. In the present study, gene expression of FACS was lower in fish fed the high-fat diet, which may reduce the β-oxidation capacity. FA transport, from ingestion to β-oxidation, correlates with β-oxidation [65]. FABP and FATP contribute to the uptake and transport of FAs throughout the cytoplasm [65]. The elevated gene expression of these two molecules in fish fed a high-fat diet indicates increased FA uptake and transport, which correlates with increased lipid accumulation. Although the muscle and adipose tissues also play important roles in fish lipid metabolism. In this manuscript, we mainly investigated the effects of a fat-rich diet on the mechanisms of fat deposition in the liver. Therefore, the gene expressions were only determined in liver.

In conclusion, our results demonstrate that reduced CPT I and ACO activities largely contribute to the attenuated hepatic β-oxidation capacity observed in fish fed a high-fat diet. Enzyme-kinetics analysis revealed that in fish fed a high-fat diet, CPT I has a low affinity for its substrates and a low catalytic efficiency. Low expression levels of the *CPT I* and *ACO* genes, and of the transcription factor PPARα, may decrease the activities of CPT I and ACO. Changes in the FA composition of the mitochondrial membrane may alter the kinetics of CPT I. Our ultrastructural and biochemical findings suggest that fish fed a high-fat diet had mitochondrial lesions, which may also negatively affect CPT I function. Overall, decreased activity and/or catalytic efficiency of the rate-limiting enzymes CPT I and ACO are mainly responsible for the impaired β-oxidation capacity in fish fed a high-fat diet.

## Supporting Information

Table S1 **Sequences and primers for genes real time PCR detecting.**
(XLSX)
